# Roles of Host Immunity in Viral Myocarditis and Dilated Cardiomyopathy

**DOI:** 10.1155/2018/5301548

**Published:** 2018-05-07

**Authors:** Lifang Zhao, Zhaoying Fu

**Affiliations:** ^1^Affiliated Hospital, Yan'an University, Yan'an, Shaanxi Province 716000, China; ^2^College of Medicine, Yan'an University, Yan'an, Shaanxi Province 716000, China; ^3^Institute of Molecular Biology and Immunology, Yan'an University, Yan'an, Shaanxi Province 716000, China

## Abstract

The pathogenesis of viral myocarditis includes both the direct damage mediated by viral infection and the indirect lesion resulted from host immune responses. Myocarditis can progress into dilated cardiomyopathy that is also associated with immunopathogenesis. T cell-mediated autoimmunity, antibody-mediated autoimmunity (autoantibodies), and innate immunity, working together, contribute to the development of myocarditis and dilated cardiomyopathy.

## 1. Introduction

The International Society and Federation of Cardiology of the World Health Organization (WHO) defined myocarditis in 1995 as an inflammatory disease of the heart muscle, diagnosed by established histological, immunological, and immunohistochemical criteria [1]. Most cases of myocarditis are of viral origin [2–4]. Many viruses have been implicated as causes of myocarditis, including coxsackieviruses group B, parvoviruses, echoviruses, adenovirus, influenza virus H1N1, Epstein–Barr virus, rubella (German measles) virus, varicella (chickenpox) virus, mumps virus, measles virus, yellow fever virus, dengue fever virus, polio virus, rabies virus, hepatitis A and C viruses, human immunodeficiency virus (HIV), and Zika virus; while parvovirus B19 (PVB19) has recently be demonstrated by endomyocardial biopsy (combined with polymerase chain reaction and in situ hybridization) as the most frequently detected virus in myocarditis, coxsackievirus B3 (CVB3) remains the most extensively studied virus that causes myocarditis both in human beings and in animal models [5–10]. Viral myocarditis usually progresses on two stages although the exact pathophysiology mechanism in humans is still not completely understood: first, the viral infection generates direct damages to the myocardium (virus-mediated lysis of myocardial cells), and then host immune responses produce indirect lesions of the cardiac muscle by killing virus-infected (antiviral immunity) and uninfected (autoimmunity) cardiomyocytes; some cases progress to dilated cardiomyopathy, and some may result in heart failure or sudden death [11–15]. Researchers found several years ago in animal experiments that the infection of BALB/c mice with coxsackievirus B3 (an RNA virus) and murine cytomegalovirus (a DNA virus) led to essentially the same pathophysiological outcomes in the heart [16]; in addition, immunosuppressive and immunoadsorption therapies have been reported to alleviate symptoms and ameliorate heart function in myocarditis and dilated cardiomyopathy patients [17–20]; these results strongly suggested the importance of the immunopathological process in the disease(s). The roles of host immunity in the development of viral myocarditis and in dilated cardiomyopathy are summarized.

## 2. T Cell-Mediated Autoimmunity

In cell-mediated immune responses, T cells are the most important immune-competent cells, and they also play a crucial role in the pathogenesis of viral myocarditis. Woodruff and Woodruff [21] first established the implication of T cells in the pathogenesis of this disease using CD1 and BALB/c mice in 1974. They demonstrated that pretreatment of CD1 mice with rabbit anti-thymocyte serum greatly suppressed the production of inflammation and tissue injury in the hearts after CVB3 infection, and deprivation of T cells by thymectomy and lethal irradiation led to a decrease in mortality and a decrease in cardiac inflammation and necrosis in CVB3-infected BALB/c mice. Following that, numerous researches were conducted to demonstrate the involvement of T cells in the pathogenesis of viral myocarditis and to determine T cell subtypes that take part in the immunopathogenesis. Kishimoto et al. [22] examined the changes in percentages of T and B cells in peripheral blood and the heart of DBA/2 mice inoculated with encephalomyocarditis virus. They found a marked decrease in T cell number in peripheral blood on day 14 but no significant changes in B cell number throughout the entire period. T cells accounted for about 80% of the cells in the myocardial tissue on days 7 and 14. Huber and Pfaeffle [23] found that the Th1 cell response required the activation of *γ*/*δ* T cells. In addition, male and female BALB/c mice differ in response to CVB3 infection. The viral infection resulted in substantial infiltration of inflammatory cells and lymphocytes in the myocardium; while male mice gave predominantly a Th1 cell response, female mice gave predominantly a Th2 cell response [24].

Later, Opavsky et al. [25] used knockout mice lacking an individual component of the T cell receptor (TCR) or coreceptor (CD4^−/−^, CD8^−/−^, CD4^−/−^, and CD8^−/−^, or TCR*β*^−/−^) to observe the contribution of T cell subpopulations to host susceptibility to CVB3 myocarditis. The disease was more severe in CD8^−/−^ mice but reduced in CD4^−/−^ mice. Removal of both CD4 and CD8 molecules from T cells by genetic knockout protected the mice from the disease. In TCR*β*^−/−^ (T cell receptor *β* chain knockout) mice, prolonged survival and minimal myocardial lesion were observed after CVB3 infection. In CD4^−/−^CD8^−/−^ mice, increased interferon-*γ* (IFN-*γ*) and decreased tumor necrosis factor-*α* (TNF-*α*) expression and reduced myocardial damage were observed. These results indicated that the presence of TCR*αβ*^+^ T cells could boost host susceptibility to viral myocarditis. One mechanism by which CD4^+^ and CD8^+^ T cell subsets mediate the pathogenesis of viral myocarditis may be related to the expression of specific cytokines. The findings in the mice with genetical CD4 and/or CD8 deficiency supported the conclusion that the cellular inflammatory infiltration following viral infection in susceptible mouse strains contributes substantially to the mortality and myocardium lesion associated with viral infection [26].

Increasing evidence supports the earlier findings that myocarditis is an autoimmune disease that involves the participation of CD4^+^ T cells [27–36]. The activation of T cells requires costimulatory signals and the respective binding of CD28 and CD40 ligands on the surface of T cells to B7 and CD40 molecules on the surface of antigen-presenting cells; otherwise, the T cells will be in a state of anergy [37–44]. Matsui et al. [45] using transgenic technology effectively prevented the transmission of costimulatory signals and thus greatly reduced the severity of experimental autoimmune myocarditis, confirming that the activation of T cells could promote the development of viral myocarditis. More and more researches demonstrated that the cognate interaction between the inducible costimulatory molecule (ICOS) and ICOS ligand (ICOSL), a member of the CD28 family, is an indispensable signaling for the activation of T cells. Blocking the ICOS–ICOSL signaling with anti-ICOS antibodies can block or attenuate myocarditis resulting from autoimmunity [46–49].

Most recently, the role of Th17 cells in viral myocarditis and dilated cardiomyopathy has drawn much attention [50–53]. The Th17 cell is a CD4^+^ T cell subpopulation distinct from IFN-*γ*-producing Th1 and IL-4-producing Th2 and is characterized by secreting IL-17, a proinflammatory cytokine [54]; many studies over the past decade have been focusing on IL-17 and Th17 cell participation in the inflammatory process of the autoimmune diseases. Earlier studies in EAM showed that IL-17 might be the critical effector cytokine responsible for EAM because neutralization of IL-17 could reduce myocarditis and heart autoantibody responses and that IL-17 promoted the recruitment of monocytes, the major heart-infiltrating cells in EAM, to the heart [55]. Yuan et al. [56] reported that IL-17 was related to the progression of acute viral myocarditis (AVMC) in a mouse model through regulating autoantibody production and neutralization of IL-17 could inhibit autoantibody production in CVB3-induced AVMC. In the CVB3-induced AVMC mouse model, Yuan et al. [57] also showed that Th17 cells contributed to viral replication and that IL-17 was important for the regulation. Using IL-17 monoclonal antibody-treated viral myocarditis mice, Fan et al. [58] showed that IL-17 was critically complicated in the pathogenesis of murine viral myocarditis and inhibition of IL-17 could alleviate the myocardium inflammation. Zhu et al. [59] reported that inhibition of Th17 cells by the newly discovered cytokine IL-27 could effectively ameliorate CVB3-induced viral myocarditis. Myers et al. [60] recently identified a Th17 cell phenotype of human myocarditis/dilated cardiomyopathy that had raised CD4^+^ IL-17^+^ T cells and Th17-promoting cytokines including IL-6, IL-23, and TGF-*β* as well as GM-CSF (granulocyte-macrophage colony-stimulating factor) secreting CD4^+^ T cells. They found that the Th17 phenotype was associated with the effects of cardiac myosin on CD14^+^ monocytes and heart failure. Persistent heart failure was linked with high proportions of IL-17-producing T cells and IL-17-promoting cytokines and the phenotype contained within a significantly low proportion of FOXP3^+^ Tregs; these may be related to disease severity.

Studies pointed out [61] that Th17 cell differentiation is mediated by the interaction between the costimulatory signal CD28 and ICOS, but interaction between cytotoxic T-lymphocyte-associated protein 4 (CTLA-4) and B7 inhibits Th17 cell differentiation. Martín et al. [62] showed that the C-type lectin receptor CD69 inhibited Th17 cell differentiation by promoting the activation of the Jak3 signal transducer and activator of the transcription 5 signaling pathway. IL-23 is required for Th17 cell's maintenance and pathogenic function. The IL-23/Th17 pathway is involved in the pathogenesis of several autoimmune diseases. Using a CVB3-induced murine model of viral myocarditis, Yang et al. [63] showed that the IL-23/Th17 axis is involved in the viral myocarditis. A study by Yamashita et al. [64] showed that IL-6 mediated Th17 differentiation in the onset (but not the progression) of EAM through ROR*γ*t, an isoform of retinoic acid receptor-related orphan receptor, and ROR*γ*t has been suggested as a master regulator for Th17 differentiation. Lately, Liu et al. [65] showed that microRNA-21 and -146b are related to the pathogenesis of viral myocarditis in mice through regulation of Th17 differentiation. Using microarrays, the authors detected the upregulation of miRNA-21 and -146b in a murine model of viral myocarditis. Silencing of miRNA-21 and -146b reduced ROR*γ*t expression, decreased Th17 level, and ameliorated the severity of viral myocarditis.

It has been well established that CD80 (or B7-1) and CD86 (or B7-2) are crucial costimulatory molecules in T cell activation, inducing Th1 and Th2 differentiation, respectively, in host immune responses. Most recently, Huang et al. [66] investigated the role of CD80 and CD86 in Th17 differentiation in AVMC. The authors infected C57BL/6 mice with CVB3 and examined its effects on Th17 differentiation with anti-CD80 and anti-CD86 monoclonal antibodies (McAbs). The results revealed that treatment with anti-CD80 McAb induced significant suppression of Th17 differentiation and ROR*γ*t mRNA expression both in vivo and in vitro, while anti-CD86 McAb treatment did not show such effect. Thus, it is CD80 not CD86 that regulates Th17 cell differentiation.

Regulatory T cells (Treg cells or Tregs) belong to a subset of CD4 T cells that express the biomarkers FOXP3 and CD25 in addition to CD4 and function to keep immune homeostasis by suppressing the development of effector T cells in particular Th17 cells that participate in the pathogenesis of a number of autoimmune diseases by producing IL-17 which promotes inflammation [67–70]. The balance between the immunosuppressive Treg cells and the proinflammatory Th17 cells is very important in host immune responses, and an imbalance between them plays a critical role in many inflammatory and autoimmune diseases. It has been established that the differentiation and proliferation of Treg cells are controlled by the transcriptional factor Forkhead box protein P3 (Foxp3) in combination with transforming growth factor beta (TGF-*β*), and the immunosuppressive function of Tregs is depending on the anti-inflammatory cytokines IL-10 and IL-35, as well as TGF-*β*, while the differentiation and proliferation of Th17 cells are regulated by the transcriptional factor ROR*γ*t in combination with IL-6 and the function of Tregs is dependent on the proinflammatory cytokines IL-17, IL-21, and IL-22. The interleukin-1 family member 7 or IL-37, the seventh member of the IL-1 family of eleven members, has recently been recognized as one of the few anti-inflammatory cytokines and is capable of suppressing a wide spectrum of proinflammatory responses. An et al. [71] reported that IL-37 suppressed Th17 response and enhanced Treg response in the spleen of a CVB3-induced murine viral myocarditis model. IL-37 downregulated the expression of Th17-related cytokines IL-17 and IL-6 but upregulated the expression of Treg-related cytokine IL-10 in the murine heart. Thus, IL-37 may exert an anti-inflammatory function in the mouse model of viral myocarditis through mediating a balance between Th17 cells and Treg cells,

Using an EAM mouse model, Yan et al. [72] revealed that the expression miR-155, a type of microRNA that is closely related to the immune system, was greatly upraised in CD4^+^ T cells and in the cardiac muscle tissue of the EAM mice; meanwhile, there was a proliferative and functional imbalance between the Tregs and Th17 cells resulting from the active induction and proliferation of Th17 cells and an elevated resistance of Th17 cells to Treg-exerted suppression. On the other hand, inhibition of miR-155 in EAM mice resulted in lessened disease severity and cardiac damage, attenuated Th17 immune response, and reduced secretion of Th17-polarizing cytokines by dendritic cells. These findings demonstrated that miR-155 could promote the development of EAM by driving an imbalance between Tregs and Th17 cells that favors the development of Th17 cells.

## 3. Antibody-Mediated Autoimmunity

Studies have demonstrated that autoantibodies play an important role in the pathogenesis of myocarditis and in dilated cardiomyopathy [73–80]. Passive transfer of anti-heart autoantibodies can induce myocarditis and dilated cardiomyopathy in experimental animals while removal of autoantibodies with immunoadsorption or other methods can improve cardiac function of myocarditis and dilated cardiomyopathy patients [81–88]. Autoantibodies may be produced by molecular mimicry, in which viral proteins possess homologous amino acid sequences with cardiomyocyte proteins (or the virus and the host share the same antigenic determinant or epitope) and the induction of an immune response to the viral antigen thus leads to a cross-reaction with self-antigens (the antibodies produced against the viral antigen bind to or react with antigens of the cardiac muscle cells that share the same antigenic determinant with the viral antigen), resulting in autoimmunity. Alternatively, autoantibodies may be generated through the initial damage to myocardial cells by viral infection that releases a large quantity of self-antigens into the circulation, in which case the viral infection is followed by a second phase of immune process of the disease, recruiting active immunocompetent cells into the cardiac tissue that favor B cell activation and subsequent autoantibody production, resulting in myocardium damage and progression of heart disease [89–115].

A wide spectrum of autoantibodies associated with human or murine myocarditis has been described in the literature, of which the most important one is directed against the contractile protein myosin [116] (For an inclusive list of the autoantibodies, please refer to Dörner et al. [74], page 334, Table 1.) The presence of the cardiac myosin-specific autoantibodies in mice with myocarditis was first described in 1987 by Rose et al. [117], Alvarez et al. [118], and Neu et al. [119]. Neu et al. [120] reported that the infection of a susceptible mouse strain with CVB3 resulted in myocarditis associated with a high titer of myosin autoantibody specific for the cardiac myosin isoform. The injection of cardiac myosin itself in certain strains of mice also produces severe myocarditis with high titers of cardiac myosin autoantibodies. The mouse strains that are resistant to cardiac myosin induced-myocarditis did not develop increased cardiac myosin autoantibodies. On the other hand, injection of the mice with skeletal muscle myosin did not produce the expected effect, suggesting that the immunogenic epitopes or determinants are specific to the cardiac myosin isoform.

In human beings, the *α*-myosin isoform exists exclusively in cardiac myocytes, whereas the *β*-myosin isoform is found both in cardiac myocytes and in slow skeletal muscle cells. Pummerer et al. [121] mapped the pathogenic epitopes on the myosin molecules in 1996. They found that *α*-myosin is the dominant immunogenic isoform that induces myocarditis (with high severity and prevalence) whereas *β*-myosin seldom causes the disease. So, the immunogenic epitopes of *α*-myosin (amino acid sequences) must reside in regions different from *β*-myosin. Three immunogenic amino sequences were identified. One sequence (AA 614–643) located in the head of the *α*-myosin heavy chain induced severe myocarditis, whereas two others (AA 735–747 and AA 947–960) that reside in the rod of the *α*-myosin heavy chain only induced minor pathologic changes in BALB/c mice. The autoimmunogenic epitope is located in a different region in A/J mice: between amino acid residue 334 and 352 of the *α*-myosin molecule [122]. Schwimmbeck et al. [123] identified the immunogenic epitopes of human myosin using synthetic peptides. More than 44% of the seral samples from patients with myocarditis or dilated cardiomyopathy bound to a region corresponding to amino acids 345 to 352 of the human myosin heavy chain, while only 4% of the sera from healthy controls reacted with this peptide. Circulating autoantibodies to whole myosin molecules were detected in 26%–46% of the patients who had myocarditis or dilated cardiomyopathy; in these patients, the autoimmune reactivity did not show significant difference between *α*-myosin and *β*-myosin [124–126].

Caforio et al. [127] investigated whether anti-heart autoantibodies are directly pathogenic to the host by passive transfer of affinity-purified anti-heart autoantibodies from sera of patients with myocarditis to normal BALB/c mice to induce experimental myocarditis. The results showed that myocarditis was present in 52% of the mice that received purified sera from patients; in contrast, only 2% of the control mice were complicated with the disease. Yuan et al. [128] examined whether the immune tolerance to swine cardiac myosin could protect BALB/c mice that have myosin-induced myocarditis from myocardial injury. The results showed that myocardial degeneration, necrosis, and inflammatory cell infiltration were found in the nontolerance mouse group but not in the immune tolerance group, suggesting the protective effect of immune tolerance on the development of autoimmune myocarditis.

Lately, the role of *β*1-adrenoreceptor autoantibodies in the pathogenesis of dilated cardiomyopathy attracted much attention [129–139]. Dilated cardiomyopathy is a common cause of heart failure which remains a main health problem because of its high prevalence and the sudden cardiac death. The cardiopathogenic role played by autoantibodies directed against *β*1-adrenoreceptors has been established in the last two decades. Experimental mouse models have shown that *β*1-adrenoreceptor autoantibodies caused progressive dilated cardiomyopathy. Clinical studies also revealed that *β*1-adrenoreceptor autoantibodies are frequently detected in sera from dilated cardiomyopathy patients and are closely associated with the disease. Autoantibodies with *β*-adrenergic effects were first isolated from sera of idiopathic dilated cardiomyopathy patients [140]. Not long after, the presence of autoantibodies (a *γ*-globulin) against the *β*1 adrenoreceptor in sera of idiopathic dilated cardiomyopathy patients was confirmed [141].

The first step in the development of idiopathic dilated cardiomyopathy might be a viral myocarditis. Several viruses might be implicated in the pathogenesis of dilated cardiomyopathy [142]. One hypothesis was that the destruction of cardiac myocytes releases the *β*1 adrenoreceptor as an autoantigen that induces the autoimmune response. An alternative hypothesis was that *β*1-adrenoreceptor autoantibodies are produced in antimicrobial immune responses as the microbes have cross-reacting antigens with the receptor. Levin and Hoebecke [143] recently put forward a “bystander” explanation. Certain viruses, bacteria, and fungi have sequence similarity with the second extracellular loop of the *β*1 adrenoreceptor. Nearly all sequences show homology with either the epitope targeted by *β*1-adrenoreceptor autoantibodies detectable in Chagas disease patients or the epitope recognized by *β*1-adrenoreceptor autoantibodies from idiopathic dilated cardiomyopathy patients. In consideration of the fact that the microbial flora of the human intestinal tract as well as most of the symbiotic microorganisms habiting in the human body are mostly unknown, it is most possibility that similar or common epitopes are present among them and that under pathological conditions these epitopes may be presented to the adaptive immune system and induce pathological immune responses [143].

Compared with autoantibody-negative cases, dilated cardiomyopathy patients who are positive in *β*1-adrenoreceptor autoantibodies showed a poorer left ventricle function, a higher occurrence of severe ventricular arrhythmias, and a higher incidence of sudden cardiac death [144]. It was found that the existence of stimulating *β*1-adrenoreceptor autoantibodies is independently associated with a roughly threefold increase in cardiac death risk in dilated cardiomyopathy patients [145]. The pathogenic role of *β*1-adrenoreceptor autoantibodies was confirmed in animal experiments in which peptides corresponding to the second extracellular loop of *β*1 adrenoreceptors could trigger similar changes in the myocardium to those observed in dilated cardiomyopathy patients [146]. Some researchers have proposed that even though *β*1-adrenoreceptor autoantibodies are not the only autoantibodies detectable in sera of dilated cardiomyopathy patients, they tended to play a more important part in the initiation and development of dilated cardiomyopathy than did other autoantibodies [147].

## 4. Innate Immunity in Viral Myocarditis

Innate immune responses also play an important role in the development of myocarditis and are responsible for the progression to dilated cardiomyopathy. The roles of Toll-like receptors (TLRs) are discussed below.

When host cells are faced with pathogens, pattern recognition receptors (PRRs), most importantly TLRs, recognize pathogen-associated molecular patterns (PAMPs) and activate an intracellular network of signaling pathways that results in the production of numerous cytokines that may exert protection and may also cause inflammation [148–151].

Of all PRRs, TLRs are first described and the most intensively studied. A total of thirteen TLRs (TLR1 through TLR13) have been identified, of which the latter two (TLR12 and TLR13) are not found in humans [152–155]. TLRs are able to recognize different PAMPs presented on various microorganisms including viruses, bacteria, and fungi [156–164]. In addition to immune cells, TLRs are expressed in various tissues, in particular in the myocardial tissue; the latter may account for the links between the immune system and cardiac injury [165–167].

TLR3, which recognizes viral double-stranded RNA, is important in the early response to virus infection. Hardarson et al. [168] examined the role of TLR3 in protection from encephalomyocarditis virus (EMCV) infection. They infected TLR3-deficient (TLR3^−/−^) mice with EMCV. These TLR3^−/−^ mice were more susceptible to EMCV infection and had a much higher viral load in their heart tissue than TLR3^+/+^ mice. Histopathological examination indicated that myocardial inflammation was less obvious in TLR3^−/−^ mice than in TLR3^+/+^ mice. TLR3^−/−^ mice also produced less proinflammation cytokines and chemokines after EMCV infection. Gorbea et al. [169] reported that individuals carrying mutated TLR3 (genetic variant P554S or L412F) had a reduced innate immune response to enteroviruses and increased viral replication through a mechanism involving inhibited NF-*κ*B and type I interferon signaling, which diminished viral clearance and increased the chance of cardiac pathology. Gorbea et al. [170] also found that depletion of extracellular mutant 29 (Ecm29), an adaptor protein of a proteasome subset, increased the abundance of TLR3 in HEK-293 cells and in HeLa cells. The absence of Ecm29 increased TLR3 signaling, increased phosphorylation/activation of effector kinases downstream of TLR3, and enhanced nuclear localization of interferon regulatory factor 3 (IRF3) and the building up of signaling molecules in juxtanuclear recycling endosomes. Thus, Ecm proteasomes are related to the trafficking of TLR3 and the attenuation of TLR3-dependent signaling.

TLR4, the first TLR to be found in human beings, has been reported to perform a variety of functions in a number of pathological conditions, including myocarditis, and its level in the heart is the highest compared with other TLRs [171–173]. TLR4 recognizes lipopolysaccharide (LPS), which leads to the activation of numerous transcription factors via two signaling pathways: MYD88- (myeloid differentiation primary response 88-) dependent pathway and MYD88-independent pathway [174–177].

Studies have shown that inhibition of the TLR4 system could reduce the severity of myocardial inflammation. Fairweather et al. [178] studied the effects of TLR4 deficiency in CVB3 infection and myocarditis. They found that the severity of myocarditis, degree of viral replication, and levels of IL-1*β*/IL-18 expression were significantly reduced in TLR4-deficient mice and that TLR4 as well as IL-12R*β*1 aggravated CVB3 infection and myocarditis but IFN-*γ* inhibited viral replication. TLR4 and IL-12R*β*1 might share common downstream pathways that directly affected IL-1*β* and IL-18 production, and IL-1beta and IL-18 played an important part in the pathogenesis of CVB3-induced myocarditis.

Fuse et al. [179] examined the role of MYD88, an important adaptor protein in the TLR4 signaling pathway, in the pathogenesis of CVB3-induced myocarditis. They found that the MYD88 level in cardiac tissue was increased significantly in wild-type mice after infection of CVB3. MYD88^−/−^ mice showed a significantly higher survival rate than did MYD88^+/+^ mice after exposure to CVB3. Pathological examination displayed a significant decrease of heart inflammation in MYD88^−/−^ mice. Cardiac viral concentrations were significantly decreased in MYD88^−/−^ mice. The levels of mRNAs for IL-1*β*, TNF-*α*, IFN-*γ*, and IL-18 were significantly decreased in the heart of MYD88^−/−^ mice, and serum levels of Th1 cytokines were significantly decreased in these mice as well. By contrast, cardiac levels of activated IRF3 and IFN-*β* (but not other usual upstream signals of IRF3) were significantly increased in these MYD88^−/−^ mice. These results indicated that MYD88 may be a very important mediator in cardiac inflammation, inducing cytokine production and maintaining Th1/Th2 cytokine balance. Deficiency of MYD88 may provide protection to the host heart through direct activation of IRF3 and IFN-*β*.

## 5. Differing Perspectives or Interpretations

### 5.1. Roles of CD4^+^ Regulatory T Cells

CD4^+^ and CD8^+^ T cells have been reported to be involved in the pathogenesis of myocarditis by many authors; however, regulatory T cells (Tregs), a subset of CD4^+^ T cells, have been shown to have protective effects [180, 181]. Shi et al. [181] recently demonstrated that the adoptive transfer of Treg cells protected the mice intraperitoneally challenged with CVB3 from myocarditis through the TGF-*β*–CAR (transforming growth factor *β*-coxsackie virus and adenovirus receptor) pathway, which maintained the antiviral immune response against CVB3 and thus suppressed the immune response to the cardiac tissue.

### 5.2. Viruses as Pathogens or Passengers

A large number of viruses have been detected in cardiac tissue, but the interpretation of their role in myocarditis is controversial. Nielsen et al. [182] examined the prevalence of three strains of viruses (adenovirus, enterovirus, and PVB19) in myocardial autopsy specimens from deceased individuals with myocarditis and in noninflammatory control hearts. They found that adenovirus, enterovirus, and PVB19 were rare causes of myocarditis. The detection of PVB19 in myocardial autopsy specimens, in particular, most likely represents a persistent infection with no or limited association with myocardial inflammation.

## 6. Summary

Immune responses in viral myocarditis function as a double-edged sword: it may be beneficial to the host by limiting viral spread and eliminating the viruses; nevertheless, excessive immune responses can damage cardiac muscle cells and contribute to destructive consequences which could lead to dilated cardiomyopathy. The boundary between the protective antiviral effects and the harmful immunopathological process is usually not clear [183–186]. Careful clinical and laboratory examinations could help doctors to make good judgment and to choose proper medications.

## References

[1] Richardson P., McKenna W., Bristow M. (1996). Report of the 1995 World Health Organization/International Society and Federation of Cardiology Task Force on the definition and classification of cardiomyopathies.

[2] Caforio A. L. P., Malipiero G., Marcolongo R., Iliceto S. (2017). Myocarditis: a clinical overview.

[3] Sagar S., Liu P. P., Cooper L. T. (2012). Myocarditis.

[4] Cooper L. T. (2009). Myocarditis.

[5] Fairweather D., Stafford K. A., Sung Y. K. (2012). Update on coxsackievirus B3 myocarditis.

[6] Spartalis M., Tzatzaki E., Spartalis E., Damaskos C., Mavrogeni S., Voudris V. (2017). Parvovirus B19 myocarditis of fulminant evolution.

[7] Verdonschot J., Hazebroek M., Merken J. (2016). Relevance of cardiac parvovirus B19 in myocarditis and dilated cardiomyopathy: review of the literature.

[8] Lobo M. L. S., Taguchi Â., Gaspar H. A., Ferranti J. F., Carvalho W. B. d., Delgado A. F. (2014). Fulminant myocarditis associated with the H1N1 influenza virus: case report and literature review.

[9] Ntusi N. A. B. (2017). HIV and myocarditis.

[10] Minhas A. M., Nayab A., Iyer S. (2017). Association of Zika virus with myocarditis, heart failure, and arrhythmias: a literature review.

[11] Fung G., Luo H., Qiu Y., Yang D., McManus B. (2016). Myocarditis.

[12] Kindermann I., Barth C., Mahfoud F. (2012). Update on myocarditis.

[13] Javadi B., Sahebkar A. (2017). Natural products with anti-inflammatory and immunomodulatory activities against autoimmune myocarditis.

[14] Weintraub R. G., Semsarian C., Macdonald P. (2017). Dilated cardiomyopathy.

[15] Nikolaou M., Lazaros G., Karavidas A., Hatzianastasiou S., Miliopoulos D., Adamopoulos S. (2017). Recurrent viral myocarditis: the emerging link toward dilated cardiomyopathy.

[16] Rose N. R. (2016). Viral myocarditis.

[17] Lu C., Qin F., Yan Y., Liu T., Li J., Chen H. (2016). Immunosuppressive treatment for myocarditis: a meta-analysis of randomized controlled trials.

[18] Camargo P. R., Okay T. S., Yamamoto L., del Negro G. M. B., Lopes A. A. (2011). Myocarditis in children and detection of viruses in myocardial tissue: implications for immunosuppressive therapy.

[19] Felix S. B., Beug D., Dörr M. (2015). Immunoadsorption therapy in dilated cardiomyopathy.

[20] Pei J., Li N., Chen J. (2012). The predictive values of beta_1_-adrenergic and M_2_ muscarinic receptor autoantibodies for sudden cardiac death in patients with chronic heart failure.

[21] Woodruff J. F., Woodruff J. J. (1974). Involvement of T lymphocytes in the pathogenesis of coxsackie virus B_3_ heart disease.

[22] Kishimoto C., Kuribayashi K., Masuda T., Tomioka N., Kawai C. (1985). Immunologic behavior of lymphocytes in experimental viral myocarditis: significance of T lymphocytes in the severity of myocarditis and silent myocarditis in BALB/c-nu/nu mice.

[23] Huber S. A., Moraska A., Choate M. (1992). T cells expressing the gamma delta T-cell receptor potentiate coxsackievirus B3-induced myocarditis.

[24] Huber S. A., Pfaeffle B. (1994). Differential Th1 and Th2 cell responses in male and female BALB/c mice infected with coxsackievirus group B type 3.

[25] Opavsky M. A., Penninger J., Aitken K. (1999). Susceptibility to myocarditis is dependent on the response of *αβ* T lymphocytes to coxsackieviral infection.

[26] Knowlton K. U., Badorff C. (1999). The immune system in viral myocarditis: maintaining the balance.

[27] Bracamonte-Baran W., Čiháková D., Sattler S., Kennedy-Lydon T. (2017). Cardiac autoimmunity: myocarditis.

[28] Gao X., Wei B., Deng Y., Huang Y. L., Wu W. (2017). Increased mobilization of CD45+CD34+VLA-4+ cells in acute viral myocarditis induced by coxsackievirus B3.

[29] Comarmond C., Cacoub P. (2017). Myocarditis in auto-immune or auto-inflammatory diseases.

[30] Tajiri K., Yasutomi Y., Aonuma K. (2016). Recent advances in the management of autoimmune myocarditis: insights from animal studies.

[31] Valaperti A. (2016). Drugs targeting the canonical NF-*κ*B pathway to treat viral and autoimmune myocarditis.

[32] Muller A. M., Fischer A., Katus H. A., Kaya Z. (2015). Mouse models of autoimmune diseases - autoimmune myocarditis.

[33] Reddy J., Massilamany C., Buskiewicz I., Huber S. A. (2013). Autoimmunity in viral myocarditis.

[34] Leuschner F., Katus H. A., Kaya Z. (2009). Autoimmune myocarditis: past, present and future.

[35] Rose N. R. (2009). Myocarditis: infection versus autoimmunity.

[36] Afanasyeva M., Rose N. (2004). Viral infection and heart disease: autoimmune mechanisms.

[37] Hui E., Cheung J., Zhu J. (2017). T cell costimulatory receptor CD28 is a primary target for PD-1–mediated inhibition.

[38] Tian R., Wang H., Gish G. D. (2015). Combinatorial proteomic analysis of intercellular signaling applied to the CD28 T-cell costimulatory receptor.

[39] Maltzman J. S., Turka L. A. (2013). T-cell costimulatory blockade in organ transplantation.

[40] Lichtman A. H. (2012). T cell costimulatory and coinhibitory pathways in vascular inflammatory diseases.

[41] Ito T., Ueno T., Clarkson M. R. (2005). Analysis of the role of negative T cell costimulatory pathways in CD4 and CD8 T cell-mediated alloimmune responses in vivo.

[42] Vu M. D., Li X. C., Ludewig B., Hoffmann M. W. (2005). Study of T-cell costimulatory blockade in vivo at a single-cell level.

[43] Najafian N., Khoury S. J. (2003). T cell costimulatory pathways: blockade for autoimmunity.

[44] Sho M., Sandner S. E., Najafian N. (2002). New insights into the interactions between T-cell costimulatory blockade and conventional immunosuppressive drugs.

[45] Matsui Y., Inobe M., Okamoto H. (2002). Blockade of T cell costimulatory signals using adenovirus vectors prevents both the induction and the progression of experimental autoimmune myocarditis.

[46] Marisa L., Svrcek M., Collura A. (2018). The balance between cytotoxic T-cell lymphocytes and immune checkpoint expression in the prognosis of colon tumors.

[47] Mo L., Chen Q., Zhang X. (2017). Depletion of regulatory T cells by anti-ICOS antibody enhances anti-tumor immunity of tumor cell vaccine in prostate cancer.

[48] Brzoza Z., Grzeszczak W., Trautsolt W., Moczulski D. (2017). Inducible T-cell costimulator (*ICOS*) and *CD28* polymorphisms possibly play a role in the pathogenesis of chronic autoreactive urticaria.

[49] Futamatsu H., Suzuki J., Kosuge H. (2003). Attenuation of experimental autoimmune myocarditis by blocking activated T cells through inducible costimulatory molecule pathway.

[50] Long Q., Liao Y. H., Xie Y. (2016). Coxsackievirus B3 directly induced Th17 cell differentiation by inhibiting Nup98 expression in patients with acute viral myocarditis.

[51] Jin H., Guo X. (2016). Valproic acid ameliorates coxsackievirus-B3-induced viral myocarditis by modulating Th17/Treg imbalance.

[52] Li L., Li L., Xiao L., Shangguan J. (2018). Progranulin ameliorates coxsackievirus-B3-induced viral myocarditis by downregulating Th1 and Th17 cells.

[53] Cheng H., Xi Y., Chi X., Wu Y., Liu G. (2016). Fenofibrate treatment of rats with experimental autoimmune myocarditis by alleviating Treg/Th17 disorder.

[54] Barin J. G., Baldeviano G. C., Talor M. V. (2013). Fatal eosinophilic myocarditis develops in the absence of IFN-*γ* and IL-17A.

[55] Valaperti A., Marty R. R., Kania G. (2008). CD11b^+^ monocytes abrogate Th17 CD4^+^ T cell-mediated experimental autoimmune myocarditis.

[56] Yuan J., Yu M., Lin Q. W. (2010). Neutralization of IL-17 inhibits the production of anti-ANT autoantibodies in CVB3-induced acute viral myocarditis.

[57] Yuan J., Yu M., Lin Q. W. (2010). Th17 cells contribute to viral replication in coxsackievirus B3-induced acute viral myocarditis.

[58] Fan Y., Weifeng W., Yuluan Y., Qing K., Yu P., Yanlan H. (2011). Treatment with a neutralizing anti-murine interleukin-17 antibody after the onset of coxsackievirus b3-induced viral myocarditis reduces myocardium inflammation.

[59] Zhu H., Lou C., Liu P. (2015). Interleukin-27 ameliorates coxsackievirus-B3-induced viral myocarditis by inhibiting Th17 cells.

[60] Myers J. M., Cooper L. T., Kem D. C. (2016). Cardiac myosin-Th17 responses promote heart failure in human myocarditis.

[61] Ying H., Yang L., Qiao G. (2010). Cutting edge: CTLA-4–B7 interaction suppresses Th17 cell differentiation.

[62] Martín P., Sánchez-Madrid F. (2011). CD69: an unexpected regulator of T_H_17 cell–driven inflammatory responses.

[63] Yang F., Wu W. F., Yan Y. L., Pang Y., Kong Q., Huang Y. L. (2011). Expression of IL-23/Th17 pathway in a murine model of coxsackie virus B3-induced viral myocarditis.

[64] Yamashita T., Iwakura T., Matsui K. (2011). IL-6-mediated Th17 differentiation through ROR*γ*t is essential for the initiation of experimental autoimmune myocarditis.

[65] Liu Y. L., Wu W., Xue Y. (2013). MicroRNA-21 and -146b are involved in the pathogenesis of murine viral myocarditis by regulating TH-17 differentiation.

[66] Huang Y., Li Y., Wei B., Wu W., Gao X. (2017). CD80 regulates Th17 cell differentiation in coxsackie virus B3-induced acute myocarditis.

[67] Dawson N. A. J., Vent-Schmidt J., Levings M. K. (2017). Engineered tolerance: tailoring development, function, and antigen-specificity of regulatory T cells.

[68] Yu H., Paiva R., Flavell R. A. (2017). Harnessing the power of regulatory T-cells to control autoimmune diabetes: overview and perspective.

[69] Kraj P., Ignatowicz L. (2017). The mechanisms shaping the repertoire of CD4^+^ Foxp3^+^ regulatory T cells.

[70] Kitagawa Y., Sakaguchi S. (2017). Molecular control of regulatory T cell development and function.

[71] An B., Liu X., Li G., Yuan H. (2017). Interleukin-37 ameliorates coxsackievirus B3-induced viral myocarditis by modulating the Th17/regulatory T cell immune response.

[72] Yan L., Hu F., Yan X. (2016). Inhibition of microRNA-155 ameliorates experimental autoimmune myocarditis by modulating Th17/Treg immune response.

[73] Simpson K. E., Cunningham M. W., Lee C. K. (2016). Autoimmunity against the heart and cardiac myosin in children with myocarditis.

[74] Dörner A., Kallwellis-Opara A., Pauschinger M., Kühl U., Schultheiss H. P. (2005). Cardiac autoantibodies in viral myocarditis.

[75] Caforio A. L. P., Tona F., Bottaro S. (2008). Clinical implications of anti-heart autoantibodies in myocarditis and dilated cardiomyopathy.

[76] Yoshikawa T., Baba A., Nagatomo Y. (2009). Autoimmune mechanisms underlying dilated cardiomyopathy.

[77] Latva-Hirvelä J., Kytö V., Saraste A. (2009). Development of troponin autoantibodies in experimental coxsackievirus B3 myocarditis.

[78] Matsumori A. (2012). Lessons learned from experimental myocarditis.

[79] Maisch B., Ristić A. D., Hufnagel G., Pankuweit S. (2002). Pathophysiology of viral myocarditis: the role of humoral immune response.

[80] Saygili E., Noor-Ebad F., Schröder J. W. (2015). Autoantibodies in dilated cardiomyopathy induce vascular endothelial growth factor expression in cardiomyocytes.

[81] Bhardwaj G., Dörr M., Sappa P. K. (2017). Endomyocardial proteomic signature corresponding to the response of patients with dilated cardiomyopathy to immunoadsorption therapy.

[82] Dandel M., Wallukat G., Englert A., Hetzer R. (2013). Immunoadsorption therapy for dilated cardiomyopathy and pulmonary arterial hypertension.

[83] Trimpert C., Herda L. R., Eckerle L. G. (2010). Immunoadsorption in dilated cardiomyopathy: long-term reduction of cardiodepressant antibodies.

[84] Becker N. P., Müller J., Göttel P., Wallukat G., Schimke I. (2017). Cardiomyopathy — an approach to the autoimmune background.

[85] Wallukat G., Müller J., Haberland A. (2016). Aptamer BC007 for neutralization of pathogenic autoantibodies directed against G-protein coupled receptors: a vision of future treatment of patients with cardiomyopathies and positivity for those autoantibodies.

[86] Haberland A., Holtzhauer M., Schlichtiger A. (2016). Aptamer BC 007 – a broad spectrum neutralizer of pathogenic autoantibodies against G-protein-coupled receptors.

[87] Dandel M., Englert A., Wallukat G. (2015). Immunoadsorption can improve cardiac function in transplant candidates with non-ischemic dilated cardiomyopathy associated with diabetes mellitus.

[88] Pokrovsky S. N., Ezhov M. V., Safarova M. S. (2013). Ig apheresis for the treatment of severe DCM patients.

[89] Benvenga S., Guarneri F. (2016). Molecular mimicry and autoimmune thyroid disease.

[90] Rose N. R. (2014). Learning from myocarditis: mimicry, chaos and black holes.

[91] Massilamany C., Koenig A., Reddy J., Huber S., Buskiewicz I. (2016). Autoimmunity in picornavirus infections.

[92] Root-Bernstein R., Fairweather D. (2015). Unresolved issues in theories of autoimmune disease using myocarditis as a framework.

[93] Root-Bernstein R. (2014). Rethinking molecular mimicry in rheumatic heart disease and autoimmune myocarditis: laminin, collagen IV, CAR, and B1AR as initial targets of disease.

[94] Massilamany C., Huber S. A., Cunningham M. W., Reddy J. (2014). Relevance of molecular mimicry in the mediation of infectious myocarditis.

[95] Massilamany C., Gangaplara A., Steffen D., Reddy J. (2011). Identification of novel mimicry epitopes for cardiac myosin heavy chain-*α* that induce autoimmune myocarditis in A/J mice.

[96] Huber S. A. (2006). Autoimmunity in coxsackievirus B3 induced myocarditis.

[97] Fujinami R. S., von Herrath M. G., Christen U., Whitton J. L. (2006). Molecular mimicry, bystander activation, or viral persistence: infections and autoimmune disease.

[98] Paleev F. N., Kotova A. A., Suchkov S. V., Paleev N. R. (2002). Modern aspects of immunopathogenesis of autoimmune myocarditis.

[99] Lawson C. M. (2000). Evidence for mimicry by viral antigens in animal models of autoimmune disease including myocarditis.

[100] Davies J. M. (1997). Molecular mimicry: can epitope mimicry induce autoimmune disease?.

[101] Gauntt C. J., Tracy S. M., Chapman N. (1995). Coxsackievirus-induced chronic myocarditis in murine models.

[102] Gauntt C. J., Arizpe H. M., Higdon A. L. (1995). Molecular mimicry, anti-coxsackievirus B3 neutralizing monoclonal antibodies, and myocarditis.

[103] Maisch B., Bauer E., Cirsi M., Kochsiek K. (1993). Cytolytic cross-reactive antibodies directed against the cardiac membrane and viral proteins in coxsackievirus B3 and B4 myocarditis. Characterization and pathogenetic relevance.

[104] Gauntt C., Higdon A., Bowers D., Maull E., Wood J., Crawley R. (1993). What lessons can be learned from animal model studies in viral heart disease?.

[105] Lawson C. M., O'Donoghue H. L., Reed W. D. (1992). Mouse cytomegalovirus infection induces antibodies which cross-react with virus and cardiac myosin: a model for the study of molecular mimicry in the pathogenesis of viral myocarditis.

[106] Beisel K. W., Srinivasappa J., Prabhakar B. S. (1991). Identification of a putative shared epitope between Coxsackie virus B4 and alpha cardiac myosin heavy chain.

[107] Aranda-Uribe I. S., Ortega E., Martínez-Cordero E. (2017). Immunization of BALB/c mice with pigeon IgY induces the production of anti-IgG autoantibodies.

[108] Kerr J. R. (2016). The role of parvovirus B19 in the pathogenesis of autoimmunity and autoimmune disease.

[109] Suurmond J., Diamond B. (2015). Autoantibodies in systemic autoimmune diseases: specificity and pathogenicity.

[110] Backes C., Ludwig N., Leidinger P. (2011). Immunogenicity of autoantigens.

[111] Barzilai O., Ram M., Shoenfeld Y. (2007). Viral infection can induce the production of autoantibodies.

[112] Nakamura H., Kawakami A., Eguchi K. (2006). Mechanisms of autoantibody production and the relationship between autoantibodies and the clinical manifestations in Sjögren’s syndrome.

[113] Paque R. E., Miller R. (1992). Adoptively transferred anti-idiotype pulsed B cells mediate autoimmune myocarditis.

[114] Siloşi I., Siloşi C. A., Boldeanu M. V. (2016). The role of autoantibodies in health and disease.

[115] Ma W. T., Chang C., Gershwin M. E., Lian Z. X. (2017). Development of autoantibodies precedes clinical manifestations of autoimmune diseases: a comprehensive review.

[116] Nussinovitch U., Shoenfeld Y. (2013). The clinical and diagnostic significance of anti-myosin autoantibodies in cardiac disease.

[117] Rose N. R., Beisel K. W., Herskowitz A. (1987). Cardiac myosin and autoimmune myocarditis.

[118] Alvarez F. L., Neu N., Rose N. R., Craig S. W., Beisel K. W. (1987). Heart-specific autoantibodies induced by Coxsackievirus B_3_: identification of heart autoantigens.

[119] Neu N., Beisel K. W., Traystman M. D., Rose N. R., Craig S. W. (1987). Autoantibodies specific for the cardiac myosin isoform are found in mice susceptible to coxsackievirus B3-induced myocarditis.

[120] Neu N., Rose N. R., Beisel K. W., Herskowitz A., Gurri-Glass G., Craig S. W. (1987). Cardiac myosin induces myocarditis in genetically predisposed mice.

[121] Pummerer C. L., Luze K., Grässl G. (1996). Identification of cardiac myosin peptides capable of inducing autoimmune myocarditis in BALB/c mice.

[122] Donermeyer D. L., Beisel K. W., Allen P. M., Smith S. C. (1995). Myocarditis-inducing epitope of myosin binds constitutively and stably to I-Ak on antigen-presenting cells in the heart.

[123] Schwimmbeck P. L., Bland N. K., Schultheiss H. P., Strauer B. E. (1991). The possible value of synthetic peptides in the diagnosis and therapy of myocarditis and dilated cardiomyopathy.

[124] Caforio A. L., Grazzini M., Mann J. M. (1992). Identification of alpha- and beta-cardiac myosin heavy chain isoforms as major autoantigens in dilated cardiomyopathy.

[125] Wang Z., Liao Y., Dong J., Li S., Wang J., Fu M. L. (2003). Clinical significance and pathogenic role of anti-cardiac myosin autoantibody in dilated cardiomyopathy.

[126] Latif N., Baker C. S., Dunn M. J., Rose M. L., Brady P., Yacoub M. H. (1993). Frequency and specificity of antiheart antibodies in patients with dilated cardiomyopathy detected using SDS-PAGE and western blotting.

[127] Caforio A. L. P., Angelini A., Blank M. (2015). Passive transfer of affinity-purified anti-heart autoantibodies (AHA) from sera of patients with myocarditis induces experimental myocarditis in mice.

[128] Yuan H. T., Liao Y. H., Wang Z. (2003). Prevention of myosin-induced autoimmune myocarditis in mice by anti-L_3_T_4_ monoclonal antibody.

[129] Müller J., Wallukat G., Schimke I. (2017). Autoantibodies directed against the *β*_1_-adrenergic receptor in patients with dilated cardiomyopathy.

[130] Park M., Reddy G. R., Wallukat G., Xiang Y. K., Steinberg S. F. (2017). *β*
_1_-Adrenergic receptor O-glycosylation regulates N-terminal cleavage and signaling responses in cardiomyocytes.

[131] Nagatomo Y., McNamara D. M., Tang W. H. W. (2017). Reply: understanding subclass diversity of detectable *β*_1_-adrenergic receptor autoantibodies and their clinical impact.

[132] Gao W., Guo W. J., Hou D. Y. (2017). Autoantibodies against *β*_1_-adrenergic receptor: response to induction therapy with bortezomib-containing regimens for multiple myeloma patients.

[133] Lei Z. X., Li B. T., Li H. A. (2017). Relationship between catecholamine level and gene polymorphism of *β*1 adrenergic receptor G1165C in children with EV71 infection in hand foot and mouth disease.

[134] Hayashi K., Gotou M., Matsui T., Imahashi K., Nishimoto T., Kobayashi H. (2017). Identification of phosphorylation sites on *β*1-adrenergic receptor in the mouse heart.

[135] Magvanjav O., McDonough C., Gong Y. (2017). Pharmacogenetic associations of *β*1-adrenergic receptor polymorphisms with cardiovascular outcomes in the SPS3 trial (Secondary Prevention of Small Subcortical Strokes).

[136] Vanhoutte L., Guilbaud C., Martherus R. (2017). MRI assessment of cardiomyopathy induced by *β*1-adrenoreceptor autoantibodies and protection through *β*3-adrenoreceptor overexpression.

[137] Patel J. K. (2017). The *β*_1_-adrenergic receptor IgG subclass 3 autoantibody in dilated cardiomyopathy: *Friend or Foe?*.

[138] Bornholz B., Hanzen B., Reinke Y., Felix S. B., Boege F. (2016). Impact of common *β*_1_-adrenergic receptor polymorphisms on the interaction with agonistic autoantibodies in dilated cardiomyopathy.

[139] Dalal S., Connelly B., Singh M., Singh K. (2018). NF2 signaling pathway plays a pro-apoptotic role in *β*-adrenergic receptor stimulated cardiac myocyte apoptosis.

[140] Wallukat G., Wollenberger A. (1987). Effects of the serum gamma globulin fraction of patients with allergic asthma and dilated cardiomyopathy on chronotropic beta adrenoceptor function in cultured neonatal rat heart myocytes.

[141] Limas C. J., Goldenberg I. F., Limas C. (1989). Autoantibodies against beta-adrenoceptors in human idiopathic dilated cardiomyopathy.

[142] Maekawa Y., Ouzounian M., Opavsky M. A., Liu P. P. (2007). Connecting the missing link between dilated cardiomyopathy and viral myocarditis: virus, cytoskeleton, and innate immunity.

[143] Levin M. J., Hoebeke J. (2008). Cross-talk between anti-*β*_1_-adrenoceptor antibodies in dilated cardiomyopathy and Chagas’ heart disease.

[144] Iwata M., Yoshikawa T., Baba A., Anzai T., Mitamura H., Ogawa S. (2001). Autoantibodies against the second extracellular loop of beta_1_-adrenergic receptors predict ventricular tachycardia and sudden death in patients with idiopathic dilated cardiomyopathy.

[145] Störk S., Boivin V., Horf R. (2006). Stimulating autoantibodies directed against the cardiac *β*_1_-adrenergic receptor predict increased mortality in idiopathic cardiomyopathy.

[146] Jahns R., Boivin V., Hein L. (2004). Direct evidence for a *β*_1_-adrenergic receptor-directed autoimmune attack as a cause of idiopathic dilated cardiomyopathy.

[147] Lappé J. M., Pelfrey C. M., Tang W. H. W. (2008). Recent insights into the role of autoimmunity in idiopathic dilated cardiomyopathy.

[148] Ohto U. (2017). Conservation and divergence of ligand recognition and signal transduction mechanisms in toll-like receptors.

[149] O'Neill L. A. J., Golenbock D., Bowie A. G. (2013). The history of toll-like receptors — redefining innate immunity.

[150] Kono D. H., Baccala R., Theofilopoulos A. N. (2013). TLRs and interferons: a central paradigm in autoimmunity.

[151] Mohammad Hosseini A., Majidi J., Baradaran B., Yousefi M. (2015). Toll-like receptors in the pathogenesis of autoimmune diseases.

[152] Jiménez-Dalmaroni M. J., Gerswhin M. E., Adamopoulos I. E. (2016). The critical role of toll-like receptors — from microbial recognition to autoimmunity: a comprehensive review.

[153] Dong L. N., Wang J. P., Liu P., Yang Y. F., Feng J., Han Y. (2017). Faecal and mucosal microbiota in patients with functional gastrointestinal disorders: correlation with toll-like receptor 2/toll-like receptor 4 expression.

[154] Shah M., Anwar M. A., Kim J. H., Choi S. (2016). Advances in antiviral therapies targeting toll-like receptors.

[155] Achek A., Yesudhas D., Choi S. (2016). Toll-like receptors: promising therapeutic targets for inflammatory diseases.

[156] Farrugia M., Baron B. (2017). The role of toll-like receptors in autoimmune diseases through failure of the self-recognition mechanism.

[157] Ntoufa S., Vilia M. G., Stamatopoulos K., Ghia P., Muzio M. (2016). Toll-like receptors signaling: a complex network for NF-*κ*B activation in B-cell lymphoid malignancies.

[158] Paarnio K., Väyrynen S., Klintrup K. (2017). Divergent expression of bacterial wall sensing Toll-like receptors 2 and 4 in colorectal cancer.

[159] Guo Y., Chai Q., Zhao Y., Li P., Qiao J., Huang J. (2015). Increased activation of toll-like receptors-7 and -8 of peripheral blood mononuclear cells and upregulated serum cytokines in patients with pediatric systemic lupus erythematosus.

[160] Zhang J., Zhou J., Xu B., Chen C., Shi W. (2015). Different expressions of TLRs and related factors in peripheral blood of preterm infants.

[161] Qiao J., Luan B., Gu H., Zhang Y. (2015). Effect of different 1, 25-(OH)2D3 doses on high mobility group box1 and toll-like receptors 4 expression in lung tissue of asthmatic mice.

[162] Tiwari A., Singh P., Jaitley P. (2017). *Eucalyptus robusta* leaves methanolic extract suppresses inflammatory mediators by specifically targeting TLR4/TLR9, MPO, COX2, iNOS and inflammatory cytokines in experimentally-induced endometritis in rats.

[163] Feng L., Yang N., Li C. (2017). Pudilan xiaoyan oral liquid alleviates LPS-induced respiratory injury through decreasing nitroxidative stress and blocking TLR4 activation along with NF-ΚB phosphorylation in mice.

[164] Chen Y., Li H., Li M. (2017). *Salvia miltiorrhiza* polysaccharide activates T lymphocytes of cancer patients through activation of TLRs mediated -MAPK and -NF-*κ*B signaling pathways.

[165] Frantz S., Ertl G., Bauersachs J. (2007). Mechanisms of disease: Toll-like receptors in cardiovascular disease.

[166] Guo X., Xue M., Li C. J. (2016). Protective effects of triptolide on TLR4 mediated autoimmune and inflammatory response induced myocardial fibrosis in diabetic cardiomyopathy.

[167] Adamczak D. M. (2017). The role of toll-like receptors and vitamin d in cardiovascular diseases—a review.

[168] Hardarson H. S., Baker J. S., Yang Z. (2007). Toll-like receptor 3 is an essential component of the innate stress response in virus-induced cardiac injury.

[169] Gorbea C., Makar K. A., Pauschinger M. (2010). A role for Toll-like receptor 3 variants in host susceptibility to enteroviral myocarditis and dilated cardiomyopathy.

[170] Gorbea C., Rechsteiner M., Vallejo J. G., Bowles N. E. (2013). Depletion of the 26S proteasome adaptor Ecm29 increases Toll-like receptor 3 signaling.

[171] Yang Y., Lv J., Jiang S. (2016). The emerging role of Toll-like receptor 4 in myocardial inflammation.

[172] Zuliani-Alvarez L., Marzeda A. M., Deligne C. (2017). Mapping tenascin-C interaction with toll-like receptor 4 reveals a new subset of endogenous inflammatory triggers.

[173] Yang J., Zhang R., Jiang X. (2017). Toll-like receptor 4–induced ryanodine receptor 2 oxidation and sarcoplasmic reticulum Ca^2+^ leakage promote cardiac contractile dysfunction in sepsis.

[174] Lopes Pires M. E., Clarke S. R., Marcondes S., Gibbins J. M. (2017). Lipopolysaccharide potentiates platelet responses via toll-like receptor 4-stimulated Akt-Erk-PLA_2_ signalling.

[175] Jang J. C., Li J., Gambini L. (2017). Human resistin protects against endotoxic shock by blocking LPS–TLR4 interaction.

[176] Zhang F. X., Xu R. S. (2018). Juglanin ameliorates LPS-induced neuroinflammation in animal models of Parkinson’s disease and cell culture via inactivating TLR4/NF-*κ*B pathway.

[177] Cochet F., Peri F. (2017). The role of carbohydrates in the lipopolysaccharide (LPS)/toll-like receptor 4 (TLR4) signalling.

[178] Fairweather D., Yusung S., Frisancho S. (2003). IL-12 receptor *β*1 and toll-like receptor 4 increase IL-1*β*- and IL-18-associated myocarditis and coxsackievirus replication.

[179] Fuse K., Chan G., Liu Y. (2005). Myeloid differentiation factor-88 plays a crucial role in the pathogenesis of Coxsackievirus B3–induced myocarditis and influences type I interferon production.

[180] Marchant D. J., McManus B. M. (2010). Regulating viral myocarditis: allografted regulatory T cells decrease immune infiltration and viral load.

[181] Shi Y., Fukuoka M., Li G. (2010). Regulatory T cells protect mice against coxsackievirus-induced myocarditis through the transforming growth factor *β*–coxsackie-adenovirus receptor pathway.

[182] Nielsen T. S., Hansen J., Nielsen L. P., Baandrup U. T., Banner J. (2014). The presence of enterovirus, adenovirus, and parvovirus B19 in myocardial tissue samples from autopsies: an evaluation of their frequencies in deceased individuals with myocarditis and in non-inflamed control hearts.

[183] Huber S. A. (2016). Viral myocarditis and dilated cardiomyopathy: etiology and pathogenesis.

[184] Sinagra G., Anzini M., Pereira N. L. (2016). Myocarditis in clinical practice.

[185] Krejci J., Mlejnek D., Sochorova D., Nemec P. (2016). Inflammatory cardiomyopathy: a current view on the pathophysiology, diagnosis, and treatment.

[186] Trachtenberg B. H., Hare J. M. (2017). Inflammatory cardiomyopathic syndromes.

